# Expanding social networks through physical activity: implications for cardiometabolic health

**DOI:** 10.1093/abm/kaag063

**Published:** 2026-09-24

**Authors:** Becky Marquez, Xinlian Zhang, Dori Pekmezi, Bess Marcus

**Affiliations:** Herbert Wertheim School of Public Health & Human Longevity Science, University of California San Diego, La Jolla, CA 92093, United States; Herbert Wertheim School of Public Health & Human Longevity Science, University of California San Diego, La Jolla, CA 92093, United States; Department of Health Behavior, University of Alabama at Birmingham, Birmingham, AL 35294, United States; Department of Behavioral and Social Sciences, Brown University School of Public Health, Providence, RI 02903, United States

**Keywords:** physical activity, social networks, cardiometabolic markers, physical activity intervention, Latinas

## Abstract

**Importance:**

Both social networks (SN) and physical activity (PA) are linked to cardiovascular health, yet prospective studies examining them simultaneously are scarce.

**Objective:**

This study examined the temporal relationship between SN and PA as well as the association of both factors with cardiometabolic markers.

**Design:**

A 1-year randomized controlled trial

**Setting:**

Remote intervention recruited from San Diego County, California

**Participants:**

Community sample of underactive adult Latinas (N-199)

**Intervention:**

Cognitive-behavioral and individually tailored PA program delivered remotely

**Main Outcomes and Measures:**

The 7-day Physical Activity Recall Interview measured moderate-to-vigorous PA (MVPA) and the Social Network Index Scale measured social network size, social role diversity, close ties (eg, family and friends), and peripheral ties (eg, neighbors and coworkers) at baseline and months 6 and 12. Total cholesterol, low density lipoprotein (LDL), high density lipoprotein (HDL), and triglycerides (TG) were collected from a non-fasting blood sample at baseline and month 6. Data were analyzed using lagged mixed-effect modeling and exploratory multivariable change-score regression.

**Results:**

Participants who engaged in greater MVPA subsequently had more peripheral social ties. The reverse association was not observed, as SN did not predict later MVPA. Increased total network size, diverse ties, or peripheral ties were associated with reduced total cholesterol and LDL, but not HDL or TG, independent of concurrent changes in MVPA. No significant associations were detected between MVPA and lipid biomarkers.

**Conclusions and Relevance:**

Engaging in PA can help cultivate new social relationships. The formation of broader and acquaintance-based social connections may contribute to better cardiometabolic health.

## Introduction

Social networks (SN) can benefit cardiovascular health. Greater social connectedness reduces risk for stroke, coronary heart disease, and heart failure, particularly in women.^1^ SN can provide social support for health-promoting behaviors such as physical activity (PA).^2^ Indeed, there is evidence to suggest that PA may mediate the association between social network size and cardiovascular risk.^3^

Given the importance of PA in cardiovascular health, developing sustainable PA interventions is crucial for high-risk women. Latina women have lower PA levels which contributes to elevated risk factors (eg, obesity and diabetes) for poor cardiovascular health. To address this disparity, cost-effective programs involving remote (ie, mail, internet, and phone delivered) PA interventions have demonstrated efficacy in improving moderate-to-vigorous PA (MVPA) among Latinas.^4^^,^^5^ Because these remote and individual-level interventions do not facilitate interpersonal interactions (eg, in-person or group-based exercise sessions), they provide an ideal context to investigate how naturally occurring SN relate to changes in PA behavior. In the *Seamos Saludables* study, the PA intervention was successful at increasing support for exercise adoption from family at 6 months and maintenance from friends at 12 months.^6^ The PA intervention had an indirect effect on PA through social support. In the *Pasos Hacia la Salud* study, assessment of familial and friendship networks showed that participants with more connections to people that had provided esteem and instrumental support for exercise engaged in higher levels of MVPA at 12 months.^7^ Moreover, participants who were connected to people that exercised were more active at 12 months. Together, these studies suggest that SN influence PA adherence of intervention participants.

Whereas intervention research has focused on functional social support for PA, a growing body of observational studies have reported on the link between structural social support and PA. Functional social support refers to the qualitative aspects of social relationships such as exchange of resources (eg, emotional or practical support) whereas structural social support refers to quantitative characteristics of social relationships such as the number of social contacts (eg, network size) or frequency of social contact. Social network size has been the most commonly studied indicator of structural support in relation to PA as it captures the potential opportunity for social influence. Studies have found that larger and more diverse SN are associated with greater PA, indicating that multiple and different types of relationships may provide novel information or broader access to resources that encourage PA.^8^

Only a small number of studies have examined social network size and PA among Latinas, with findings indicating that individuals with larger SN are likely to walk more, meet leisure-time PA guidelines, and engage in higher levels of MVPA.^9^^,^^10^^,^^7^ A limitation of this literature is its predominant focus on close relationships (ie, strong ties) involving family members and friends, with comparatively less attention to peripheral relationships (ie, weak ties) such as neighbors, coworkers, or members of affiliated groups. The research gap is especially relevant for immigrant groups such as Latinas who may rely more on extrafamilial networks following migration to the United States (U.S.). Furthermore, prospective studies that concurrently assess SN and PA remain notably scarce, although there is some indication of a bidirectional association.^11^ Presumably SN can provide support for PA engagement, while PA participation can provide opportunities for social interaction. However, more research is needed to understand how these factors evolve and affect health over time.

The current study builds on prior research of SN and PA among Latinas enrolled in PA interventions. *Seamos Activas II* was a 1-year remote PA intervention targeting underactive Latina women. The intervention design offers a unique opportunity to conduct longitudinal analyses to gain insight into the interplay between SN and PA as well as their impact on cardiometabolic health. The objectives of the study are to examine the temporal relationship between SN and PA and the association of both these factors with cardiometabolic markers.

## Methods

### Participants

*Seamos Activas II* was a 1-year randomized controlled trial consisting of a cognitive-behavioral and individually-tailored PA intervention for underactive Latinas delivered via print-based mailings alone (original group) or with text messages and phone calls (enhanced group).^12^ Latino/Hispanic females aged 18-65 years old who engaged in fewer than 60 minutes/week of MVPA were recruited from the community through mass media, social media, and community outreach efforts. Participants had on average 43.8 ± 10.1 years of age and a body mass index (BMI) of 30.4 ± 5.2 kg/m^2^. Most participants had at least a high school education (71%) and were U.S. immigrants (82%). Participants in both intervention groups significantly increased minutes of weekly MVPA at 6 and 12 months, with no difference between groups. Data were collected from February 2016 to July 2019. All participants provided written informed consent. The Institutional Review Board at the University of California San Diego approved the study.

### Measures

#### Social networks

The Social Network Index Scale focuses on social roles and the number of social ties that are in contact at least once every 2 weeks from each social role.^13^ Social roles consist of close ties (spouse/partner, children, parents, parent in-laws, close relatives, and close friends) and peripheral ties (neighbors, coworkers, church members, classmates, volunteers, and group members). Social network diversity is calculated from the total number of different social roles out of a possible 12. Social network size is calculated from the total number of social ties. The scale was completed at baseline, 6 months, and 12 months.

#### Physical activity

The 7-day Physical Activity Recall Interview was used to estimate the total minutes per week of MVPA performed over the past week regardless of domain (eg, leisure-time, occupational, transportation, and household activities).^14^ The interview was administered at baseline, 6 months, and 12 months.

#### Cardiometabolic markers

Total cholesterol, low density lipoprotein (LDL), high density lipoprotein (HDL), and triglycerides (TG) were collected from a non-fasting blood sample at baseline and 6 months.

### Analysis

Data from 199 participants were analyzed. Log transformation of counts data (SN and MVPA) were performed before feeding into the statistical models to address potential right skewness in distribution (ie, extremely large data values). In all models, covariates controlled for age, education, BMI, and group assignment. To evaluate the temporal relationship between each social network characteristic and MVPA, we fit 2 lagged mixed-effects (LME) models. The first model examined whether prior social network characteristic predicted subsequent MVPA, adjusting for prior MVPA. The second model examined whether prior MVPA predicted subsequent social network characteristic, adjusting for prior social network value. Both models pooled data from baseline to 6 months and 6 to 12 months intervals. Primary analyses included all available lagged observations with complete data for variables included in each model and were estimated using maximum likelihood in the lme4 package in R. As a sensitivity analysis, we fit 3-wave cross-lagged panel models (CLPM) to evaluate reciprocal, wave-specific temporal associations between each social network characteristic and MVPA. Models were estimated using robust maximum likelihood in lavaan, with full information maximum likelihood used to incorporate all available data. To examine the associations of changes in SN and MVPA with cardiometabolic markers, we fit exploratory multivariable change-score regression models. Change in each lipid biomarker from baseline to 6 months was regressed on concurrent changes in social network characteristic and the corresponding MVPA over the same interval, adjusting for baseline biomarker value and covariates. Because changes in SN, MVPA, and lipid biomarkers were measured concurrently, these analyses were interpreted as associations rather than evidence of temporal ordering or mediation.

## Results

### Temporal relationship between SN and MVPA

Whereas prior SN did not predict subsequent MVPA, higher prior MVPA was significantly associated with greater subsequent peripheral social ties (Table 1). The magnitude of this association was modest; specifically, a doubling of prior MVPA was associated with an approximately 4% increase in subsequent peripheral ties. Cross-lagged panel models analysis further showed that the observed association was driven primarily by the 6-12 months interval, in which higher MVPA at 6 months predicted greater peripheral ties at 12 months. There was suggestive evidence of an association between social role diversity and MVPA, with greater diverse ties predicting higher subsequent MVPA and higher MVPA predicting greater subsequent diverse ties (*P* < .10). No prospective associations of either direction were observed for total network size or close social ties by either LME or CLPM.

**Table 1:** Relationship between SN and MVPA.^a^

	B	SE	*df*	*t*	*P* value
**SN predicting PA**					
**Total network size**	0.053	0.175	78	0.302	.763
**Diverse ties**	0.792	0.401	84	1.975	.052
**Close ties**	0.345	0.250	82	1.381	.171
**Peripheral ties**	0.023	0.108	97	0.218	.828
**MVPA predicting SN**					
**Total network size**	0.018	0.18	71	0.991	.325
**Diverse ties**	0.011	0.007	79	1.694	.094
**Close ties**	−0.003	0.011	74	−0.264	.790
**Peripheral ties**	0.059	0.027	90	2.191	**.031**

a The e coefficient (B), standard error (SE), degree of freedom (*df*), *t* value (*t*), and *P* value for each social network characteristic in the LME model using SN to predict future MVPA or MVPA to predict future SN. Boldface value indicates statistical significance (p≤0.05).

### Associations with lipid biomarkers

In exploratory multivariable regression analyses, we examined whether baseline-to-6 months changes for each social network characteristic and MVPA were independently associated with concurrent changes in cardiometabolic markers (Table 2). Across models, changes in SN showed the most consistent nominal associations with lipid biomarkers. Specifically, greater increases in total network size, diverse ties, and peripheral ties were associated with larger reductions in total cholesterol and LDL cholesterol, independent of concurrent changes in MVPA. In contrast, changes in SN were not consistently associated with HDL or TG. Moreover, changes in MVPA were not consistently associated with changes in any lipid biomarker after adjustment for concurrent changes in SN.

**Table 2:** Relationship between SN, MVPA, and cardiometabolic biomarkers.^a^

	Total network size	Diverse ties	Close ties	Peripheral ties
SN				
**Total cholesterol**	**B = −15.22 (4.31)** ***P* = <.001**	**B = −30.68 (12.48)** ***P* = .018**	B = −7.664 (8.81) *P* = .389	**B = −7.19** **(2.51)** ***P* = .006**
**LDL-C**	**B = −14.40 (4.13)** ***P* = .001**	**B = −37.06 (12.10)** ***P* = .004**	B = −3.157 (8.52) *P* = .713	**B = −8.98 (2.33)** ***P* = <.001**
**HDL-C**	B = −0.469 (1.63) *P* = .775	B = −0.591 (4.43) *P* = .894	B = −1.611 (3.11) *P* = .607	B = 0.421 (0.92) *P* = .649
**TG**	B = −1.854 (12.82) *P* = .886	B = 9.578 (36.40) *P* = .794	B = −11.09 (24.46) *P* = .652	B = −0.297 (6.90) *P* = .966

	Total network size	Diverse ties	Close ties	Peripheral ties
MVPA				
**Total cholesterol**	B = −0.720 (1.39) *P* = .607	B = −1.437 (1.40) *P* = .312	B = −1.094 (1.52) *P* = .476	B = −0.813 (1.15) *P* = .483
**LDL-C**	B = −0.634 (1.33) *P* = .638	B = −1.230 (1.31) *P* = .353	B = −0.876 (1.46) *P* = .554	B = −0.710 (1.02) *P* = .493
**HDL-C**	B = 0.095 (0.54) *P* = .863	B = 0.086 (0.52) *P* = .870	B = 0.084 (0.53) *P* = .877	B = 0.482 (0.43) *P* = .274
**TG**	B = −1.479 (4.17) *P* = .725	B = −2.316 (4.05) *P* = .570	B = −1.933 (4.19) *P* = .647	B = −3.772 (3.05) *P* = .222

a The coefficient (B), standard error, and *P*-value for lipid biomarkers in the exploratory multivariable change-score regression models. Boldface values indicate ­statistical significance (p≤0.05)

## Discussion

The results of a remote and individual-level PA intervention for underactive Latinas provide new insights into the dynamic interplay between SN and PA. This study is one of the first to examine the temporal association between SN and PA during the course of a PA intervention. Findings also allow a better understanding of the roles of SN and PA in cardiometabolic health.

### Association between PA and social network growth

Our findings indicate that PA engagement preceded expansion of SN. Participants who increased their PA earlier in the intervention were connected to more people later. This supports the concept of “exercise to socialize” which was based on research showing that moderate PA predicts future reduction in loneliness among middle-aged and older adults, but that loneliness does not predict subsequent PA.^15^ Hence, longitudinal evidence suggests that engagement in PA may promote social connectedness over time. The present study reveals that uptake of PA is associated with increases in peripheral social ties. A prior study found that more activity occurred when individuals interacted with peripheral ties compared to being alone or with family or close friends.^16^ Similarly, adults are more likely to participate in PA with people they have known for a shorter time, and walking for exercise has been linked to more frequent contact with neighbors.^17^^,^^18^ Peripheral ties may have particular importance for well-being as these relationships are often voluntarily chosen, potentially involving less conflict and more shared interests. Such findings highlight the potential value of PA in facilitating formation of social connections that extend beyond close, preexisting relationships.

Contrary to expectation, SN were not associated with subsequent changes in PA. Much of the existing literature has generally reported positive associations between social relationships and PA. Unlike most prior studies, which have been observational and predominately cross-sectional, the present study evaluated predictors of MVPA change over time among underactive Latina women participating in a PA intervention. In this context, intervention-related factors may have superseded the influence of SN on later PA. Also, we assessed both close and peripheral ties while previous studies have focused on family and friendship ties rather than broader social network structure. These methodological differences may contribute to the discrepant findings.

### Association of SN and lipid biomarkers

Participants whose SN grew in size, role diversity, or peripheral ties experienced reduction in total cholesterol and LDL independent of PA. These findings may be explained by the biopsychosocial stress-inflammation model which posits that psychological stress induces an inflammatory response that elevates risk for cardiovascular disease.^19^ Increased social connectivity may have enhanced feelings of belonging or social support that can help reduce cardiometabolic markers by alleviating stress.^20^ Surprisingly, SN were not related to changes in TG or HDL, and close ties as well as MVPA were not associated with changes in any lipid biomarker. Given that most participants were immigrants to the U.S., it is possible that assessment of “close ties,” operationalized as the number of family and friends in contact within the last 2 weeks, may not have adequately captured transnational ties. Additionally, the lack of association between PA and lipid biomarkers may reflect differences in mechanisms and time course through which social relationships and PA influence cardiometabolic health. Improvements in social connectedness may reduce psychosocial stress and downstream inflammatory processes, whereas exercise-related improvements in lipid biomarkers often require larger increases in PA, higher exercise intensity, or longer-term adherence.^21^^,^^22^

### Strengths

A major strength of this study is that it is among the first to examine longitudinal changes in social network composition alongside changes in PA within a remotely delivered PA intervention targeting Latina women. These findings address an important gap in the literature, as Latina experience disproportionately high rates of physical inactivity and are at increased risk for social isolation, all of which can adversely affect health.^23^^,^^24^ Remote interventions may be particularly well suited for this population because competing work and home responsibilities, as well as transportation and scheduling barriers, can limit participation in traditional in-person programs. Importantly, our findings suggest that delivering an intervention remotely does not preclude meaningful social engagement. Participants who increased their PA also expanded their networks of non-close social ties, indicating that engaging in PA may help create opportunities to develop new social connections beyond immediate family and close friends. Because peripheral ties can provide access to novel information, resources, and social opportunities that are less likely to be available through close relationships alone, these findings suggest that remotely delivered PA interventions may confer social benefits in addition to increasing PA.

Another key strength of this study is that it provides insight into the evolution of social relationships during the process of PA adoption. The findings suggest that as individuals become more physically active, they tend to develop various informal or casual connections. PA may drive change in SN through distinct pathways. One possible mechanism is direct and involves environmental change, whereby PA engagement places individuals in settings that facilitate social interactions. For example, a study of health club members found that about 30% reported socializing with people they met at the club, and those that socialized exercised more frequently.^25^ Another possible mechanism is indirect and involves psychological change, in which PA activates cognitive/affective mediators that encourage social interactions. For example, PA is associated with higher self-efficacy, self-esteem, and positive mood which may promote receptivity to others or motivation to seek others.^26^ Further studies are needed to clarify how PA fosters social relationships.

### Limitations

Interpretation of the findings should consider the study’s limitations. PA was assessed via a self-reported measure. Only MVPA was examined and results may be different for other forms of PA that are light intensity or strength based. The intervention enrolled underactive but relatively healthy women and findings may not represent the general population. Although the majority of intervention participants had overweight/obesity, they were without serious comorbidities which may have limited the range for change in cardiometabolic markers.

### Conclusion

This study highlights the potential for scalable remotely delivered health interventions to simultaneously promote PA and foster broader social integration among Latina women. PA participation may offer meaningful opportunities to establish new social connections outside one’s core network of support. Social networks may benefit cardiometabolic health.

## Transparency statement

The parent trial was pre-registered at *clinicaltrials.gov.* Clinical Trial Registration: NCT02630953. The current study involves secondary data analysis that was not registered prior to beginning data collection at *clinicaltrials.gov.* De-identified data from this study will be made available (as allowable according to institutional IRB standards) by emailing the corresponding author. Analytic code used to conduct the analyses presented in this study are not available in a public archive. They may be available by emailing the corresponding author. Materials used to conduct the study are not publicly available.
